# Food loss of perishable produce from farm to retail: evidence from tomato supply chains in South India

**DOI:** 10.1093/ajcn/nqac039

**Published:** 2022-02-14

**Authors:** Jocelyn M Boiteau, Prabhu Pingali

**Affiliations:** Tata-Cornell Institute for Agriculture and Nutrition, Cornell University, Ithaca, New York, USA; Division of Nutritional Sciences, Cornell University, Ithaca, New York, USA; Tata-Cornell Institute for Agriculture and Nutrition, Cornell University, Ithaca, New York, USA; Division of Nutritional Sciences, Cornell University, Ithaca, New York, USA; Charles H. Dyson School of Applied Economics and Management, Cornell University, Ithaca, New York, USA

**Keywords:** food loss and waste, food supply chain, loss destination, food quality, perishable vegetables, tomato, India

## Abstract

**Background:**

Reducing food loss and waste (FLW) may narrow gaps between fruit and vegetable production and recommended intake. However, FLW estimates are inconsistent due to varying estimation methods.

**Objectives:**

Using multiple estimation approaches, we examined the extent and determinants of FLW along tomato supply chains in South India, from farm to retail. We also explored tomato quality assessments.

**Methods:**

We surveyed 75 farm households and 83 tomato traders in the Chittoor district, Andhra Pradesh, and 52 vegetable traders and 50 vegetable retailers in Hyderabad, Telangana, on harvest and market days. We calculated declared FLW values using participant-reported losses to estimate the preharvest quality FLW and quantitative FLW values at the farmer, vegetable-trader, and vegetable-retailer stages. We calculated the destination FLW based on counted crates diverted to loss destinations, using participant-reported destinations (animal feed, field discard), to estimate the postharvest FLW from farm to retail. We used pile sorting with farmers to explore on-farm quality assessments.

**Results:**

The average preharvest quality FLW was 13.9% of harvested tomatoes. From farm to retail, the quantitative FLW was greatest at the postharvest, farm level. Among all harvests, the median postharvest, farm-level FLW was 0.0% (IQR, 0.0%–7.9%) using the destination FLW approach (tomatoes diverted to nonfood uses) and 2.3% (IQR, 0.0%–12.5%) using the declared FLW approach (*P* < 0.05). Among harvests with a non-zero postharvest, farm-level FLW, the median FLW was 9.1% (IQR, 2.4%–16.7%) using the destination FLW approach (tomatoes diverted to nonfood uses) and 10.0% (IQR, 2.9%–16.7%) using the declared FLW approach. Harvesting during peak season was a determinant of postharvest, farm-level and preauction, market-level FLW values. Farmers prioritize color/ripeness attributes while harvesting and tomato size while grading.

**Conclusions:**

Single-point estimates may obscure FLW patterns for perishable, indeterminate crops and depend on data collection and estimation methods. Reducing FLW of perishables requires the integration of quantitative and qualitative FLW estimation methods.

## Introduction

The current global food system continues to struggle to provide healthy diets in the setting of increasing environmental changes. Shifting towards healthier, environmentally sustainable dietary patterns will require, in part, increased consumption of healthy foods, including fruits and vegetables, improved food production practices, and food loss and waste (FLW) reductions (1). The United Nations Sustainable Development Goal (SDG) 2, zero hunger, targets agricultural production and nutrition. However, the SDG 2 targets lack coordinated action and overlook value chain actors and activities that connect food production to food consumption (2). In many global regions, there are already deficits in fruit and vegetable availability to meet dietary recommendations, particularly in sub-Saharan Africa and South Asia (3–5).

Fruits and vegetables are among the more perishable food groups and are more at risk of FLW. As part of SDG 12 (Responsible Consumption and Production), SDG target 12.3 broadly aims to halve food waste and reduce food loss by 2030. Quantitative FLW refers to a reduction in food mass or volume, whereas food quality loss and waste (also referred to as qualitative FLW) refers to the decrease in food quality (e.g., sensory, nutritional, or food safety attributes) along the food chain without a decrease of dry food matter (6). The extent of and reasons for FLW can vary widely between supply chain contexts and stages, factors across food groups, the actors involved, and seasons (7). Understanding the nature, stages, and extent of FLW is essential for FLW reduction. There is also no harmonized FLW definition. There are often inconsistencies in terms of the supply chain stages considered and when unconsumed food is counted as FLW (8). Several recent studies have used self-report or direct measurement methods to quantify region- and supply chain–specific FLW of perishable foods, finding losses concentrated at the producer level (9–11).

Our research examines the extent, stages, and determinants of FLW along perishable vegetable supply chains in South India, from farm to retail. Unique to this study, we use data collected at the harvest and market levels on harvest and market days, respectively, to compare FLW estimates after applying different final use destinations to classify FLW. Using detailed data on production, harvest, and postharvest contexts, we examine the associated determinants of FLW at the farmer stages. Finally, we explore food quality assessments to understand perspectives on desirable food quality attributes across supply chain actors.

## Methods

This study was conducted in the Chittoor district, Andhra Pradesh, and in Hyderabad city, Telangana, across tomato supply chains from farm to retail stages. Data were collected using surveys and pile-sort group discussions from January 2019 to March 2020.

### Case study context

Tomatoes are an important horticultural crop in India. Along with onions and potatoes, tomatoes are among the top three vegetables and tubers produced (12). India ranks second in global tomato production, behind China (13). Tomato production in India is primarily carried out by smallholder farmers (14). Farmers usually sell tomatoes to local aggregators or through public, state-run wholesale markets operated by Agricultural Produce Market Committees (APMCs). In urban vegetable wholesale markets, traders source a variety of vegetables, including tomatoes, from wholesale markets and sell to other urban traders and retailers. Less than 1% of tomatoes produced in India are processed (14).

Andhra Pradesh produced the most tomatoes of any Indian state in 2017–2018 (15), and the Chittoor district is a major producing district in Andhra Pradesh (16). North of the Chittoor district, Hyderabad is the sixth most populous metropolis in India (17), and is among the many cities that import tomatoes from the Chittoor district.

Farmers growing tomatoes in the Chittoor district are smallholders, with average operating land areas of 2.1 acres (18). Most of the population in the Chittoor district is involved in agriculture, either as producers (23%) or laborers (39%) (19). In this region, the peak tomato harvest occurs during April to July (14). The Madanapalle APMC in the Chittoor district is the largest tomato wholesale market in Andhra Pradesh, and is among the major tomato trading hubs in India (20). Commission agents coordinate auctions between farmers and tomato traders. In reality, commissions agents are also tomato traders themselves. Once sold, tomatoes are repacked into plastic crates and loaded onto large trucks for transport.

In Hyderabad, vegetable wholesale markets trade tomatoes sourced from within and outside of Telangana, including from Madanapalle. It takes 10 to 12 hours for a truck loaded with tomatoes to travel the 550 km from the Madanapalle market to Hyderabad. Vegetables arrive to the wholesale market before sunrise. Trading is usually finished by late morning or early afternoon. Both traditional and modern vegetable retailers in Hyderabad purchase tomatoes from vegetable wholesale markets.

### Study participants

Among the 66 subdistricts in the Chittoor district, we purposively selected the Madanapalle and Nimmanapalle subdistricts for the study due to their close proximity to the Madanapalle tomato wholesale market. We randomly selected four *panchayats* (collections of villages) in each subdistrict. Using household rosters available from the Andhra Pradesh Horticulture Department, we then randomly selected one village per *panchayat* that listed ≥20 tomato farming households. We randomized the listed households in each village for recruitment. Households were eligible to participate if they grew tomatoes the previous year and planned to grow tomatoes during the study period. In February 2019, we aimed to enroll 15 households per village for a sample size of 120 households. After the 2019 peak tomato-harvest season, ≤33% of enrolled households from three villages/*panchayats* had tomato harvests. We expanded the study coverage area to include three additional villages, one from each low-harvesting *panchayat*, and enrolled households from July to September 2019. We used a rolling recruitment strategy to enroll tomato traders at the Madanapalle tomato market. Tomato traders who purchased tomatoes from study farm households at auction were invited to participate in the study.

In Hyderabad, three APMCs operate a total of four vegetable wholesale markets that trade tomatoes. We recruited vegetable traders from three APMC-operated wholesale markets: Bowenpally, Gudimalkapur, and Madannapeta. We purposively selected Madannapeta because, between the two markets operated by the Hyderabad APMC, Madannapeta deals with larger volumes of tomatoes. In April 2019, we carried out a census of vegetable traders to identify those who primarily deal in tomatoes, and invited all eligible vegetable traders to participate in the study. From April to July 2019, we used snowball sampling to recruit vegetable retailers, referred by study vegetable traders, and invited retailers that sell tomatoes during the study period to participate.

Informed consent was obtained from each participant prior to the survey and group discussion. This study was approved by the Cornell University Institutional Review Board (protocol ID 1810008329).

### Survey data collection

We collected data on FLW, the primary outcome, from supply chain actors across farm to retail stages. Farm household surveys included modules on initial and ongoing production activities and inputs, harvesting and marketing activities, tomato quality, and FLW. We surveyed households at the farm level on the day of harvest and, if households brought their tomatoes to the Madanapalle tomato wholesale market, at the market the day after harvest. Since tomatoes are a multiharvest crop, we used the same sets of questions through two follow-up surveys over the course of the harvesting season per plot, aiming for a total of three harvests in one plot during one harvesting season. We did not limit the number of harvesting seasons per household. Tomato-trader, vegetable-trader, and vegetable-retailer surveys included modules on marketing activities, tomato quality, and FLW. We surveyed tomato traders after they purchased tomatoes at auction from a study farmer. We surveyed vegetable traders and retailers once per month.

Across all participants, the reference period for each survey was the day of harvest or market. Vegetable-retailer surveys also included a series of questions referencing tomatoes sold on the day prior to survey. We carried out surveys in the Chittoor district from February 2019 through February 2020, and in Hyderabad from April 2019 through March 2020. We paused FLW data collection in the Chittoor district starting in January 2020 because few households were harvesting tomatoes. In February 2020, we surveyed farm households on information sources and marketing practices. In March 2020, we permanently suspended all survey data collection due to the coronavirus disease 2019 pandemic. Trained enumerators conducted in-person surveys in Telugu using the Android-based application Open Data Kit [Get ODK Inc. (getodk.org)].

**FIGURE 1:**
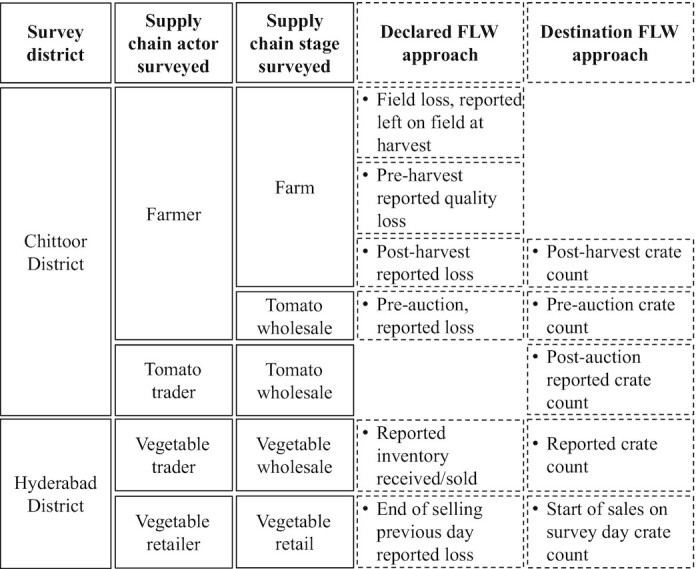
Summary of FLW data collection across survey districts, supply chain actors, and supply chain stages. As tomatoes move from farm to wholesale to retail stages, data are collected using declared FLW and destination FLW approaches. Solid lines indicate the survey context. Dashed lines indicate FLW data collected. The declared FLW approach relies on participant recall and interpretation of FLW. The destination FLW approach uses counted crates and the participant-reported destination. Examples of loss destinations include animal feed, compost, discard on-field, or trash. Abbreviations: FLW, food loss and waste.

We collected FLW data using two different approaches: participant-reported and counted crates. Without a harmonized FLW definition, there is no agreement on the criterion used for classifying unconsumed food as FLW (8). In this study context, potential destinations for diverted tomatoes included animal feed, compost, and discarded trash. We estimated FLW values based on the declared FLW and destination FLW to explore FLW estimates using different approaches and criterion. We defined the declared FLW as FLW based on participant-reported crates, which relies on the participant's recall and interpretation of classifying unconsumed food as FLW. We defined the destination FLW as FLW based on counted crates using different criterion of loss destinations. The declared FLW and destination FLW are further described below. A summary of supply chain stages and FLW data collection is presented in **Figure 1**.

We included survey questions on different food quality aspects: the market grade, important quality attributes, ripeness level, quality intensity, and price. For perceived ripeness level, we used the USDA color classification visual (21), modified with an additional ripeness level based on feedback from our study participants, similar to that of Suslow and Cantwell (22). Certain quality attributes may be correlated with others and considered as a group: firmness is closely related to ripeness, and color is most commonly used as an indicator of ripeness (23). Therefore, we asked participants to report the overall tomato quality intensity using a line scale with labeled endpoints from 1, indicating low quality, to 9, indicating high quality (24).

### Definitions used for FLW estimation approaches

We calculated the declared FLW as a percentage of the reported loss out of the reported total amount of tomatoes. Adapting questions on self-reported FLW from Delgado et al. (25), we defined field loss as harvest-ready tomatoes that are left in the field, either remaining on the plant or picked but left in the field; preharvest quality loss as quality deterioration among harvested tomatoes that occurred prior to harvest; postharvest, farm-level loss as loss among harvested tomatoes; and preauction, market-level loss as loss among tomatoes transported to the wholesale market. Vegetable traders declared FLW in terms of their inventory received and sold on survey day. Vegetable retailers declared FLW from the previous day, once sales were completed.

We estimated the destination FLW as the proportion of crates that went to a loss destination out of the total number of crates. Participants reported the final destination for each group of crates. Participants could report multiple destinations per group. Counted crates were directly observed at the farmer and retailer stages. Counted crates were reported by tomato and vegetable traders, given the large quantity of crates at these stages. When crate size was not reported, we inferred the crate size was the same as previous surveys based on multiple observations for each participant, and assuming that crate size would be time-invariant. We considered two criterion commonly used in the literature to distinguish loss destinations for food diverted away from the food supply: nonfood use and nonproductive use (7, 26, 27). Nonfood use refers to destinations where the food will not be consumed by humans, such as food diverted to animal feed or discarded on the field or at the market as trash. Nonproductive use is a narrower criterion that refers to destinations where the food will not be consumed by humans and no longer has a productive use. Nonproductive destinations include produce discarded on the field or at the market as trash. We used the prefix “any” to distinguish when participants reported destinations that fell across multiple criteria and “only” when reported destinations fell within one criterion.

### Pile-sort focus group discussions

Farmers are the first supply chain actors to assess product quality. To understand grading and sorting from farmers’ perspectives, we held pile-sort focus group discussions from January to May 2019 and in December 2019 with tomato farmers from our study villages. Four to six tomato farmers were each given a bowl of tomatoes (about 10 kg). The tomatoes were harvested on the discussion day and had not been graded or sorted. We asked participants to group tomatoes into piles of similar quality. Participants were given one opportunity to sort tomatoes before we began the group discussion. We did not constrain the number of piles to make or give any reference to specific quality criteria (28). During the discussion, participants described the sorting process and quality attributes, use and destination, and marketability for each pile. A trained staff member led the discussions in Telugu. Discussions were audio recorded with participant permission. For participants that refused to be audio recorded, we paused the recording and summarized the discussion by hand-written notes. Audio recordings were translated and transcribed into English for analysis.

### Farm-level determinants of food loss

Using evidence from existing agriculture, food science, and FLW literature, we selected independent variables a priori that are hypothesized to influence food loss outcomes via household characteristics; production, postharvest, and marketing activities and decisions; and information sources (**Supplemental Table 1**). We included caste as a covariate because caste is an indicator for several factors, including access to resources (e.g., credit), inclusion or exclusion from extension services, and regional differences in the quality of services and infrastructure (29, 30). Additionally, we included a covariate dummy variable indicating whether the household fell within the coverage area of a local nongovernmental organization that operated tomato production and marketing programs with farmers at the time of our study.

### Analytical methods and statistics

All enrolled farm households planned to harvest tomatoes during the study period, but nearly half of enrolled farm households did not have any harvests. We assessed differences between farm household characteristics that never harvested tomatoes and households that harvested at least once during the study period using the Mann–Whitney *U* and Pearson's χ^2^ tests for continuous and categorical observations, respectively. We estimated the mean and median FLW values at each supply chain stage surveyed, evaluating differences between loss estimation methods using a Kruskal–Wallis nonparametric test with a post hoc Dunn's test and Bonferroni correction.

Mixed-effects regression models were fit to explore determinants of the qualitative FLW at the preharvest stage and the FLW at the postharvest and preauction stages. We used a multi-level mixed-effects approach to account for hierarchical effects where independent variables are measured at the village, household, plot, and harvest levels (31). For postharvest and preauction loss models, we used “any, nonfood use” loss estimates. Because FLW estimates are skewed right, with a mass point at zero, we used a two-part model approach to model the dependent variable, FLW, using two steps (**Supplemental Methods**) (32). We first fit a mixed-effects binary logit model for the odds of observing a positive FLW outcome compared with a zero FLW outcome. We exponentiated both sides of the logit model to interpret the coefficients in terms of ORs. Next, conditional on a positive FLW outcome, we fit a mixed-effects linear model for the positive FLW outcome. We log-transformed the dependent variable, extent of FLW, in each linear model using the natural log to control for heteroskedasticity of the error term. We use the antilog of regression coefficients to interpret the regression results. To reduce the risk of overfitting the models, we assessed theoretically distinct sets of independent variables and constructed final models that included only significant independent variables from each set (Supplemental Table 1). Missing data varied across regression models based on the final variables included in the model, with 7% missing in the preharvest model, 11% missing in the postharvest model, and 12% missing in the preauction model. We used listwise deletion of missing data in the models.

To determine differences between market grades and quality intensity and price, we used linear regression with random effects at the participant and survey levels to account for the random variability that occurred as each participant assessed tomato quality, comparing tomatoes relative to each other on the survey day. We performed a pairwise comparison of means to determine which market grades differed from each other.

Statistical significance was set at a *P* value of 0.05. Stata (version 15.0; StataCorp) was used for all analyses. Figures were produced using R Studio version 1.3.

Pile sort interview transcripts were coded using a qualitative analysis software, ATLAS.ti (ATLAS.ti GmbH) version 8, and analyzed to understand how farmers grade and sort tomatoes. We used a deductive coding approach where we identified a coding framework a priori (33) based on our research question and theoretical framework on tomato quality and marketing (34, 35). To summarize the pile-specific patterns, we tabulated the cooccurrence of codes by code group.

## Results

### Supply chain actor characteristics

Among 145 farm households enrolled (**Supplemental Figure 1**), 48% never harvested tomatoes during the study period (**Table 1**). We observed significant differences by households with and without harvests for agriculture as a main source of income (*P* = 0.04), as well as by caste (*P* = 0.002). There was no significant difference between households and enrollment period (*P* = 0.34). Among the 56 households without harvests that were enrolled before the 2019 peak harvest season, 71% reported water scarcity as the main reason for never harvesting tomatoes. Unfortunately, no data were collected on the stage at which these farmers stopped production activities. There were no other significant differences between households for the remaining characteristics considered.

**TABLE 1:** Enrolled household characteristics by households with and without harvest^1^

	Households with harvest		Households without harvest		*P*
	*n*	Median (IQR)	*n*	Median (IQR)	
Enrolled before 2019 peak harvest season, *n*(%)	75	55 (73)	70	56 (80)	0.34^2^
Lives in area covered under NGO program,^3^*n* (%)	75	40 (53)	70	36 (51)	0.82^2^
Owned land, acres	72	3 (1.5–5.0)	67	2.5 (1.0–3.2)	0.10^4^
Leased land, acres	68	0 (0.0–1.0)	67	0.0 (0.0–0.5)	0.28^4^
Experience in tomato production, years	74	15.5 (10.0–20.0)	70	15.0 (8.0–20.0)	0.34^4^
Agriculture as a main income source, *n* (%)	75	71 (95)	69	58 (84)	0.04^2^
Member of farmer producer organization, *n* (%)	75	24 (32)	69	16 (23)	0.24^2^
Caste,^5^*n* (%)	69	—	66	—	0.002^2^
Scheduled tribe/scheduled caste	—	1 (1)	—	12 (18)	—
Lower-ranked caste^6^	—	49 (71)	—	34 (52)	—
Other caste	—	19 (28)	—	20 (30)	—

1 The sample size (*n*) changes by row due to data availability. Reported values are the median (IQR), unless otherwise indicated. Households without harvests reported their intention at enrollment to produce tomatoes during the study period, but either produced and never harvested tomatoes or never produced tomatoes. Abbreviations: NGO, nongovernmental organization.

2 *P* values from χ^2^ test.

3 A local NGO operated a tomato production and marketing program in half our study *panchayats* at the time of our study.

4 *P* values from Mann–Whitney *U* test.

5 Indian societies are stratified along the lines of several caste groups. The government of India follows affirmative action policies to correct for the historical marginalization of those at the bottom of the caste hierarchy.

6 The Indian administrative system uses “other backward class” as an official classification to denote one of the marginalized caste groups apart from the most disadvantaged scheduled castes and scheduled tribes. Additional information can be found at https://en.wikipedia.org/wiki/Other_Backward_Class and from the Ministry of Social Justice and Empowerment, Government of India, at https://socialjustice.nic.in/UserView/index?mid=31548.

We surveyed a total of 276 harvests at the farm level, of which 201 were brought to the Madanapalle wholesale market and surveyed (**Table 2**). Most households brought their harvests to the Madanapalle wholesale market at least once during the survey period. We surveyed a median of three harvests per household (IQR, 3–6 harvests). Across a total of 23 plots, 15 different households stopped harvesting tomatoes early. Farmers ended harvests early on 26% of plots (*n* = 6) because of low market prices. Additional reasons for stopping the harvests early included water scarcity, poor-quality tomatoes, and disease or pest damage. At the farm-level and market-level surveys, there was a median of one respondent per household (IQRs, 1–2 and 1–1 respondents, respectively), indicating that we typically surveyed the same household member across surveys. The majority of respondents at the farm- and market-level surveys were male, and half had at least a secondary education. Most respondents were the household head, followed by the adult child of the household head.

**TABLE 2:** Descriptive statistics of FLW surveys in the Chittoor district^1^

	Farm level	Market level
Household level		
Households surveyed, *n* (%)	75 (100)	59 (79)
Surveys per household	3 (3–6)	3 (3–6)
Respondents per household	1 (1–2)	1 (1–1)
Survey level		
Total surveys, *n*	276	201
Male respondent, *n* (%)	218 (79)	189 (94)
Respondent relationship to household head, *n* (%)		
Household head	160 (58)	129 (64)
Spouse	45 (16)	12 (6)
Adult child or child-in-law	68 (25)	52 (26)
Other relative or nonrelative	3 (1)	8 (4)
Surveys with respondent education level ≥ grade 8, *n* (%)	139 (50)	103 (51)

1 Values are median (IQR), unless otherwise indicated.

**Supplemental Table 2** summarizes tomato-trader, vegetable-trader, and vegetable-retailer characteristics. We enrolled a total of 83 tomato traders. The number of surveys given per tomato trader was skewed, averaging a mean of 2.4 surveys per trader (SD, 3.3 surveys), and a median of one survey per trader. In Hyderabad, of the 224 vegetable traders screened during the census, 78 (35%) vegetable traders were eligible and 52 traders participated (**Supplemental Figure 2**). Of the 66 vegetable retailers referred by participating vegetable traders, 61 (91%) retailers were eligible and 50 retailers participated (**Supplemental Figure 3**). Most vegetable retailers were located at daily markets (70%).

### Food loss estimates

**FIGURE 2:**
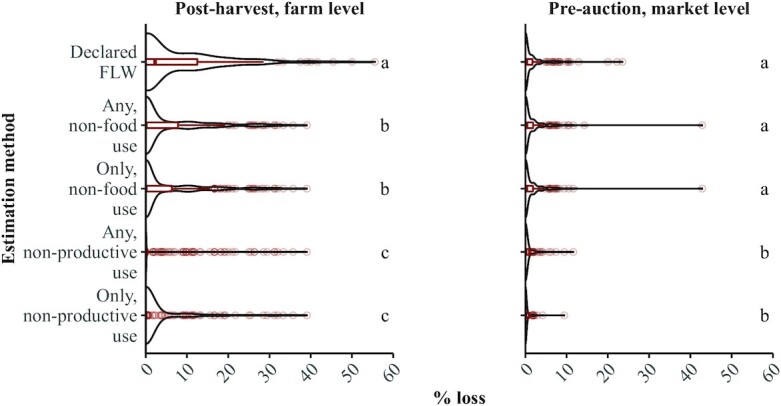
FLW estimates by estimation method and loss destination criterion at postharvest and preauction stages. Box and whisker plots in are red with a bold line indicating the median value; the box showing the 25th and 75th percentiles; the thin lines indicating the extreme line (extreme lines indicate 1.5 times the inter-quartile range from the upper or lower quartile); and the circles indicating outlying points. Violin plots are in black. Methods sharing the same letter within each panel are not significantly different (*P* > 0.05; Kruskal–Wallis/Dunn's test). Bonferroni was used to adjust for multiple comparisons. The same sample was used for destination FLW methods, representing different classification criteria of unconsumed food: “any, nonfood use” refers to tomatoes that go to at least one nonfood destination (e.g., animal feed, left on field, discarded as trash). “Only, nonfood use” refers to tomatoes that go to only nonfood destinations. “Any, nonproductive use” refers to tomatoes that go to at least one nonproductive destination (e.g., left on field, discarded as trash). “Only, nonproductive use” refers to tomatoes that go to only nonproductive destinations. Sample sizes were as follows: declared FLW method (postharvest, *n* = 234; preauction, *n* = 190), destination FLW methods (postharvest, *n* = 264; preauction, *n* = 190). Abbreviations: FLW, food loss and waste.

We report statistics on FLW estimates at the farmer stages in **Table 3** and at the wholesale and retail stages in **Supplemental Table 3**. Aggregate postharvest FLW from farm to retail totaled between 9.1% to 13.4% of the total tomato quantity, based on the destination FLW and declared FLW methods, respectively (Table 3; Supplemental Table 3). Out of 275 surveyed harvests, farmers reported that they decided to harvest based on the tomato ripeness 82% of the time and typically did not leave harvest-ready tomatoes on the field during a harvest. The majority of harvests included tomatoes that had some form of preharvest quality loss, which typically remained below 14% of harvested tomatoes among harvests with loss. Farmers reported damage from pests, disease, or animals as the major cause of preharvest quality loss over half of the time (61% of harvests with preharvest quality loss), followed by too much sun or rain or a lack of rain (21% and 10% of harvests, respectively). Over half of harvests had declared FLW at the postharvest farm level. Among harvests with losses, 9%–10% of the total harvest was lost (Table 3). The postharvest, farm-level FLW using the declared loss method was significantly different than the loss estimated using the destination loss method (**Figure 2**). Among all harvests at this stage, the median declared FLW was greater than the destination FLW using the “any, nonuse criteria” (2.3% and 0.0%, respectively; Table 3). The postharvest, farm-level FLW estimates defined by nonfood use were significantly different than the estimates defined by nonproductive use (Figure 2). For harvests brought to the Madanapalle wholesale market, 58% of harvests had some FLW at the preauction, market level. Among those with preauction FLW, typically ≤2% of the total harvest was lost (Table 3). The preauction, market-level FLW estimates using the declared method were not significantly different than the loss estimates defined by nonfood use, but were significantly different than the estimates defined by nonproductive use (Figure 2). At both the farm and market levels, tomatoes were diverted either to animal feed or discarded as trash. Farmers typically do not expect remuneration for tomatoes diverted to animal feed. Farmers reported diverting totals of 58 and 77 groups of tomatoes to animal feed at the farm and market levels, respectively, and expected to receive a median price of 0 rupees (IQR, 0–0 rupees). Farmers reported pests, disease, and animals as major causes of postharvest and preauction FLW values (73% and 49%, respectively), followed by too much sun or rain (19% and 26%, respectively).

**TABLE 3:** Food loss and waste estimates by declared and destination loss methods^1^

	Declared FLW^2^			Destination FLW^3^		
	n	Mean ± SD^4^	Median (IQR)	N	Mean ± SD	Median (IQR)
Field loss, harvest-ready tomatoes left in field						
Frequency of harvests with food loss, *n* (%)	275	20 (7)	—	—	—	—
Among all harvests, share of harvested tomatoes lost, %	—	2.0 ± 8.6	0.0 (0.0–0.0)	—	—	—
Among harvests with loss, share of harvested tomatoes lost, %	—	27.8 ± 17.9	25.0 (16.0–43.5)	—	—	—
Preharvest quality loss						
Frequency of harvests with quality loss, *n* (%)	261	180 (69)	—	—	—	—
Among all harvests, share of harvested tomatoes damaged, %	—	13.9 ± 18.9	6.7 (0.0–18.5)	—	—	—
Among harvests with loss, share of harvested tomatoes damaged, %	—	20.2 ± 19.7	13.4 (5.9–28.6)	—	—	—
Postharvest, farm level loss						
Frequency of harvests with food loss, *n* (%)	234	149 (64)	—	264^5^	121 (46)	—
Among all harvests, share of harvested tomatoes lost, %	—	7.5 ± 10.6	2.3 (0.0–12.5)	—	4.9 ± 8.4	0.0 (0.0–7.9)
Among harvests with loss, share of harvested tomatoes lost, %	—	11.9 ± 11.2	10.0 (2.9–16.7)	—	10.8 ± 9.6	9.1 (2.4–16.7)
Preauction, market level loss						
Frequency of harvests with food loss, *n* (%)	190	110 (58)	—	190	111 (58)	—
Among all harvests, share of harvested tomatoes lost, %	—	1.7 ± 3.6	0.2 (0.0–1.7)	—	1.6 ± 3.9	0.2 (0.0–1.9)
Among harvests with loss, share of harvested tomatoes lost, %	—	3.0 ± 4.3	1.5 (0.5–3.4)	—	2.8 ± 4.8	1.6 (0.5–3.4)

1 The sample size (*n*) indicates the number of harvests surveyed. The sample size changes by row due to data availability; based on field observations, common reasons for missing data were the participant did not know or, particularly at the market, the participant was not available to complete all survey questions. Abbreviation: FLW, food loss and waste.

2 FLW estimated using participant self-report.

3 FLW estimated using crate counts and considering loss destinations as “any, nonfood use.”

4 Values are mean ± SD unless otherwise indicated.

5 From 14 March to 14 April 2019, farm-level data collection was interrupted due to travel restrictions related to the 2019 Indian general election. During this period, farm-level crate counts (*n* = 11) could not be observed and are missing.

At the tomato and vegetable wholesale stages, traders typically lose <1% of their lots (Supplemental Table 3). There were no differences in FLW estimates using destination loss methods at the tomato-trader and vegetable-trader stages (data not shown). At the retail stage, vegetable retailers lose tomatoes at the start of selling when they remove tomatoes from their starting inventory; this occurred at 26% of surveys with retailers (Supplemental Table 3). Among retailers with FLW at the start of selling, a median of 2.6% (IQR, 1.5%–6.2%) of their total starting inventory went to nonfood destinations. At the start of selling, the FLW estimated using the nonfood destination criteria was significantly greater than the FLW estimated using the nonproductive destination criteria (data not shown). By the end of selling, vegetable retailers reported they had lost some of their inventory at 88% of surveys. Among retailers with FLW at the end of selling on the previous day, retailers reported they lost a median of 3.6% (IQR, 1.6%–6.0%) of their inventory (Supplemental Table 3).

### Determinants of loss at farmer stages

After adjusting for multiple covariates, the odds of preharvest quality loss occurring in harvests that took place during the peak season (April–July) were 83% lower (OR, 0.17; 95% CI: 0.04–0.75) than those of harvests during the off-peak season (**Table 4**). Because tomatoes are harvested multiple times per plot, we accounted for the harvest number reported by farmers. At least half of harvests surveyed were between the third to sixth harvest on the respective plot (**Supplemental Table 4**). Harvests with a later harvest number had odds of preharvest quality loss occurring that were 19% lower (OR 0.81; 95% CI 0.66–0.98) than those harvests with earlier harvest numbers (Table 4). Among harvests with preharvest quality loss, an increase of 100 rupees per 30 kg in expected price for tomatoes was associated with 15% less quality loss (SE = 0.04, *P <* 0.001). After adjusting for multiple covariates, the odds of a postharvest FLW occurring in harvests that took place during the peak season are 88% lower (OR, 0.12; 95% CI: 0.05–0.29) than those of harvests that took during the off-peak season (**Table 5**). Among harvests with postharvest FLW, harvesting during the peak season was associated with 63% less loss compared to harvests during the off-peak season (SE, 0.31; *P <* 0.01). Larger harvests show increased odds of postharvest FLW. For each additional 30 kg of tomatoes harvested, the odds of postharvest loss increased by 2% (OR, 1.02; 95% CI: 1.00–1.03). Among all harvests, harvests with on-farm grading and sorting had odds of postharvest FLW occurring that are 7.1 times (OR, 7.07; 95% CI: 3.31–15.10) those of harvests without on-farm grading and sorting. However, among harvests with a postharvest FLW > 0% of the total harvest, on-farm grading and sorting was associated with 51% less loss than harvests without on-farm grading (SE, 0.30; *P <* 0.05). Finally, preharvest damage was associated with postharvest FLW; among harvests with postharvest FLW, a one-percentage-point increase in preharvest damage was associated with a 2% greater postharvest loss (SE, 0.01; *P* < 0.01). Of note, 100% of farmers used plastic crates for transporting tomatoes to the market (data not shown). At the Madanapalle wholesale market, after adjusting for multiple covariates, harvests that had male family members or female hired laborers involved in either the farm-level packing or market-level grading had increased odds of preauction FLW compared to harvests without male family or female hired labor (**Table 6**). Among harvests with preauction FLW, the peak season was associated with 72% less preauction loss compared to harvests marketed during the off-peak season (SE, 0.34; *P <* 0.001).

**TABLE 4:** Estimation results from two-step mixed-effects regression models of preharvest, qualitative FLW^1^

Variables	Preharvest quality loss occurs^2^	Extent of preharvest quality loss (ln transformed)^3^	Extent of preharvest quality loss^4^
Harvest number	0.81 (0.66–0.98)^5^	—	—
Harvest season			
Off-peak (August–March)	Reference	—	—
Peak (April–July)	0.17 (0.04–0.75)^5^		
FPO member^6^	—	0.28 (0.24)	0.32
Highest price expected, 100 Rs. per 30 kg	—	−0.16 (0.04)^7^	−0.15^7^
Quality intensity, low to high quality (1–9)	—	−0.08 (0.04)	−0.08
Experience in tomato cultivation, years	—	−0.00 (0.01)	−0.00
Loss reduction strategy: applied pesticide^6^	—	0.19 (0.20)	0.21

1 All models were adjusted for caste and nongovernmental organization coverage area. Random effects are at the village, household, and plot levels. See Supplemental Table 4 for summary statistics on independent variables. Abbreviation: FLW, food loss and waste; FPO, farmer producer organization; Rs., Indian rupees.

2 Values are presented as the OR (95% CI). Mixed-effects logistic regression models are with 11 groups and 244 total observations.

3 The extent of loss was natural log-transformed. Values are presented as the β coefficient (SE). Mixed-effects linear regression models are with 10 groups and 145 total observations.

4 Because the dependent variable, extent of loss, was natural log-transformed, we took the anti-log of the β coefficient for interpretation of the coefficient in terms of the actual extent of preharvest quality loss.

5 *P* < 0.05.

6 Dummy, yes/no variable, where “no” is the reference.

7 *P* < 0.001.

**TABLE 5:** Estimation results from two-step mixed-effects regression models of postharvest, farm-level FLW^1^

Variables	Postharvest, farm-level FLW occurs^2^	Extent of postharvest, farm-level FLW (ln transformed)^3^	Extent of postharvest, farm-level FLW^4^
Harvest season			
Off-peak (August–March)	Reference	Reference	Reference
Peak (April–July)	0.12 (0.05–0.29)^5^	−0.99 (0.31)^6^	−0.63^6^
Harvesting container is a basket^7^	—	0.21 (0.28)	0.23
Area tomatoes kept during harvest			
Unshaded	Reference	—	—
Shaded	0.46 (0.21–1.05)	—	—
Container used to hold harvested tomatoes at the field			
No container	3.02 (0.67–13.54)	—	—
Plastic crate, ≤20 kg capacity	2.26 (0.73–7.03)	—	—
Plastic crate, ≥25 kg capacity	Reference	—	—
Total harvested tomatoes, 30 kg	1.02 (1.00–1.03)^8^	—	—
Grading and sorting done on-farm^7^	7.07 (3.31–15.10)^5^	–0.71 (0.30)^8^	−0.51^8^
Preharvest damage, % of harvest	1.01 (0.99–1.03)	0.02 (0.01)^6^	0.02^6^

1 All models were adjusted for caste and nongovernmental organization coverage area. Random effects were at the village, household, and plot levels. Postharvest loss estimates use the “any, nonfood use” criterion. See Supplemental Table 4 for summary statistics on independent variables. Abbreviation: FLW, food loss and waste.

2 Values are presented as OR (95% CI). Mixed-effects logistic regression models are with 11 groups and 231 total observations.

3 The extent of loss was natural log-transformed. Values are presented as β coefficient (SE). Mixed-effects linear regression models are with 11 groups and 107 total observations.

4 Because the dependent variable, extent of loss, was natural log-transformed, we took the anti-log of the β coefficient for interpretation of the coefficient in terms of the actual extent of postharvest, farm-level FLW.

5 *P* < 0.001.

6 *P* < 0.01.

7 Dummy, yes/no variable, where “no” is the reference.

8 *P* < 0.05.

**TABLE 6:** Estimation results from two-step mixed effects regression models of preauction, market level FLW^1^

	Preauction, market-level FLW occurs^2^	Extent of preauction, market-level FLW (ln transformed)^3^	Extent of preauction, market-level FLW^4^
Harvest season			
Off-peak (August–March)	Reference	Reference	Reference
Peak (April–July)	0.97 (0.39–2.41)	−1.27 (0.34)^5^	−0.72^5^
Production input: drip irrigation^6^	—	−0.87 (0.47)	−0.58
Production input: staking^6^	—	−0.92 (0.81)	−0.60
Production input: chemical fertilizer or NPK applied^6^	—	−0.79 (0.58)	−0.55
Total harvested tomatoes, 30 kg	—	0.00 (0.00)	0.00
Farm-level packing: family, male^6^	3.66 (1.11–12.03)^7^	−0.36 (0.28)	−0.30
Farm-level packing: hired, female^6^	4.90 (1.52–15.85)^8^	—	—
Market-level grading: family, male^6^	3.41 (1.08–10.78)^7^	—	—
Market-level grading: hired, female^6^	5.17 (1.65–16.27)^8^	—	—

1 All models were adjusted for caste and nongovernmental organization coverage area. Random effects are at the village, household, and plot levels. Preauction loss estimates use the “any, nonfood use” criterion. See Supplemental Table 4 for summary statistics on independent variables. Abbreviations: FLW, food loss and waste; NPK, nitrogen, phosphorus, and potassium.

2 Values are presented as OR (95% CI). Mixed-effects logistic regression models are with 10 groups and 170 total observations.

3 Values are presented as β coefficient (SE). Mixed-effects linear regression models are with 10 groups and 98 total observations.

4 Because the dependent variable, extent of loss, was natural log-transformed, we took the anti-log of the β coefficient for interpretation of the coefficient in terms of the actual extent of preauction, market-level FLW.

5 *P* < 0.001.

6 Dummy, yes/no variable, where “no” is the reference.

7 *P* < 0.05.

8 *P* < 0.01.

### Perspectives on tomato quality

**FIGURE 3:**
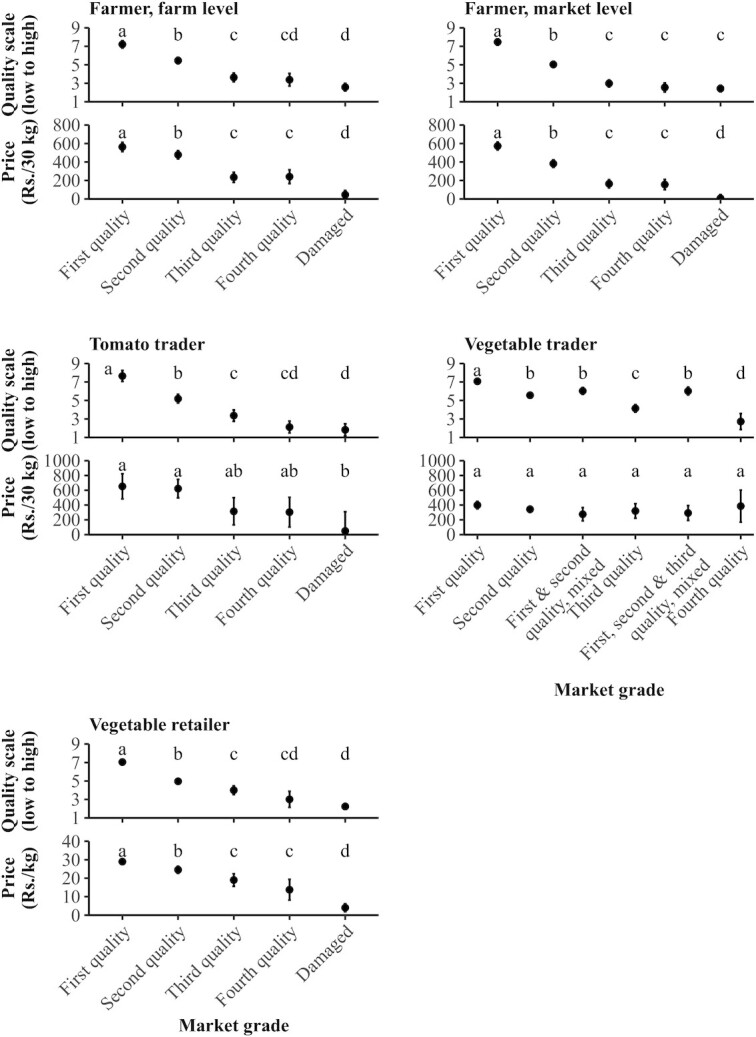
Marginal mean quality intensity and price by market grade. Farmers and tomato traders are from the Chittoor district; vegetable traders and vegetable retailers are from Hyderabad. Error bars denoted 95% CIs. Shared letters within the same panel are not significantly different at a *P* value < 0.05; analyzed by pairwise comparison with Bonferroni correction. Price refers to the price the supply chain actor expects to receive at the market. Total observations (groups of tomatoes) were as follows: farmer, farm level (Quality scale: first quality, *n* = 97; second quality, *n* = 132; third quality, *n* = 62; fourth quality, *n* = 28; damaged, *n* = 86. Price: first quality, *n* = 87; second quality, *n* = 125; third quality, *n* = 62; fourth quality, *n* = 27; damaged, *n* = 100); farmer, market level (Quality scale: first quality, *n* = 106; second quality, *n* = 110; third quality, *n* = 77; fourth quality, *n* = 45; damaged, *n* = 93. Price: first quality, *n* = 95; second quality, *n* = 102; third quality, *n* = 76; fourth quality, *n* = 44; damaged, *n* = 113); tomato trader (Quality scale: first quality, *n* = 21; second quality, *n* = 34; third quality, *n* = 20; fourth quality, *n* = 18; damaged, *n* = 17. Price: first quality, *n* = 7; second quality, *n* = 13; third quality, *n* = 6; fourth quality, *n* = 5; damaged, *n* = 3. Due to the skewed number of surveys per tomato trader, observations were collapsed to the mean per trader); vegetable trader (Quality scale: first quality, *n* = 91; second quality, *n* = 138; first and second quality, mixed, *n* = 29; third quality, *n* = 24; first, second, and third quality, mixed, *n* = 23; fourth quality, *n* = 5. Price: first quality, *n* = 91; second quality, *n* = 140; first and second quality, mixed, *n* = 29; third quality, *n* = 24; first, second, and third quality, mixed, *n* = 23; fourth quality, *n* = 5); vegetable retailer (Quality scale: first quality, *n* = 271; second quality, *n* = 129; third quality, *n* = 31; fourth quality, *n* = 9; damaged, *n* = 85. Price: first quality, *n* = 272; second quality, *n* = 130; third quality, *n* = 32; fourth quality, *n* = 10; damaged, *n* = 87). Abbreviations: Rs., Indian rupees.

Using market grade categories, we examined differences of quality intensity and expected price (**Figure 3**). Across all actors, first-, second-, and third-quality grades were significantly different from each other based on the quality scale. For farmers at the market level, damaged-quality tomatoes were not significantly different in quality scale than third-quality tomatoes; for all other stages, damaged-quality tomatoes were significantly different. At the farmer and vegetable-retailer stages, the first-, second-, third-, and damaged-quality grades were significantly different from each other based on the expected price.

**FIGURE 4:**
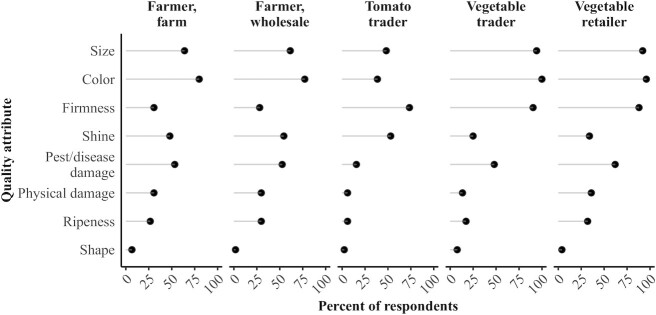
Important tomato quality attributes reported by value chain actor. Total respondents were as follows: farmer household, farm level: *n* = 75; farmer household, wholesale level: *n* = 57; tomato trader: *n* = 83; vegetable trader: *n* = 52; and vegetable retailer: *n* = 50.

We further explored important tomato quality attributes reported by each supply chain actor to better understand the quality assessments (**Figure 4**). Over half of all actors indicated size as an important attribute. Color was also important and was reported by over half of all actors other than tomato traders. Firmness was important to traders and retailers. Pest/disease damage was frequently reported among farmers and vegetable retailers, who participate in opposite ends of the supply chain.

**FIGURE 5:**
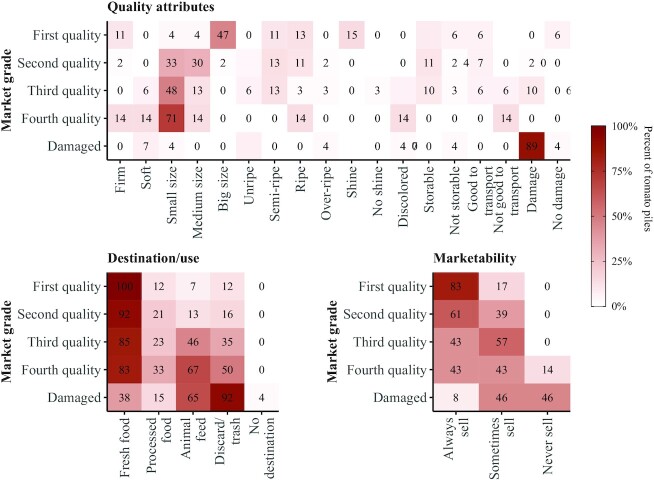
Summary of pile-sort discussion cooccurrence of quality attributes, destination/use, or marketability with market grade. Numbers in cells correspond to the percentage of tomato piles as per the color spectrum on the right. Rows do not sum to 100% because participants could mention multiple quality attributes and destinations/uses. Total piles discussed per market grade were as follows: first quality (quality attributes, *n* = 47; destination/use, *n* = 42; marketability, *n* = 42), second quality (quality attributes, *n* = 46; destination/use, *n* = 38; marketability, *n* = 36), third quality (quality attributes, *n* = 31; destination/use, *n* = 26; marketability, *n* = 28), fourth quality (quality attributes, *n* = 7; destination/use, *n* = 6; marketability, *n* = 7), and damaged (quality attributes, *n* = 27; destination/use, *n* = 26; marketability, *n* = 24).

The grouping domains, categories, and grouping aspects from the pile-sort group discussions with farmers are reported in **Supplemental Table 5**. Across 11 pile-sort group discussions, participants identified 197 tomato piles. Participants most often used market grades to refer to group quality (97% of tomato piles). While participants described both sensory and functional quality attributes (e.g., size and storability, respectively), they most often referenced the fruit size and discussed how tomatoes are downgraded as the fruit size decreases (**Figure 5**). Damaged tomatoes were typically grouped based on the presence of pest, disease, or physical damage. Most tomatoes described as at least fourth quality were reported to be used as fresh food. Participants mentioned diverting tomatoes to animal feed or discard/trash more frequently as quality worsened, with discard/trash reported for nearly all damaged tomato piles. In contrast to first-quality tomatoes that were often described as always marketable, participants only discussed never selling tomatoes when referring to fourth-quality or damaged tomatoes.

## Discussion

Opportunities exist for FLW reduction along vegetable supply chains to help close the gap between vegetable production and recommended intake and to achieve more sustainable and healthier diets (5). Particularly for perishable vegetables, factors including preharvest damage, postharvest practices, and market conditions and prices can increase FLW (6, 36). In this study of tomato supply chains in South India, food quality loss and waste occurred as early as preharvest, and most FLW occurred postharvest, before tomatoes left the farm. On harvest days, market-ready tomatoes were rarely left on the field. These findings differ from results along traditional tomato supply chains in Colombia, where farmers reported an average of 7.5% unharvested tomatoes and 13.6% unsold tomatoes during the last completed tomato crop cycle (11). Comparability with other studies is challenging because of the different loss estimation methods used, reference periods, and stages considered (10, 11, 37). When we considered different FLW estimation methods, we arrived at different estimates at the farmer and retailer stages. Postharvest, farmers reported higher FLW than was observed by counting crates for nonfood destinations; however, this difference disappeared at the preauction, market level. At the farmer and retailer stages, higher FLW estimates were observed when classifying nonfood destinations as loss, compared to nonproductive destinations. Losses were lowest at the trader stages, regardless of the destination loss method. Among the several determinants of FLW at the farmer stages, harvesting during the peak season was a significant determinant across all stages, indicating the potential importance of seasonal supply-and-demand factors on food loss as early as during the preharvest. Supply chain actors consider a number of tomato attributes when evaluating quality, primarily size and color. Farmers prioritize the color and ripeness level at the time of harvest and the fruit size at the time of grading.

Food uses and economically productive uses distinguish different criterion for classifying unconsumed food as FLW (7, 26, 27). In addition to animal feed, economically productive uses of food diverted away from human consumption include biofuel or other industrial uses, and exclude composting (7). Previous studies reporting the use of tomatoes diverted from food supply chains indicate that tomatoes are either left on the field unharvested; used as compost, for land application, or as animal feed; or discarded as trash (10, 11). In this study, tomatoes diverted from the food supply were either left in the field, used as animal feed, or discarded. As important sources of micronutrients, fruits and vegetables are produced with the clear intention for human consumption. Animal feed resources used in sustainable livestock production should not compete for human food (38). In contrast, cereal grains are produced for food and feed. Well-established secondary markets for animal feed are remunerative for producers when grain is diverted away from food supply chains. In this study, diverting tomatoes to animal feed was not remunerative for farmers or retailers. Depending on the criterion used to classify unconsumed food as FLW, loss estimates may lead to different conclusions with regard to FLW prevention and valorization, which may or may not align with all stakeholder objectives.

Tomato prices in India are often highest during the summer season (May to July), when tomato production in most of the country is in an off-season, serving as an advantage to locations with suitable summer growing conditions, like the Chittoor district (39). Cold storage is not commonly available in fresh tomato supply chains in India. Without adequate cold storage, mature produce quickly deteriorates as bruising, over-ripeness, excessive softening, and biological spoilage cause quality and postharvest losses (40, 41). Fresh tomatoes typically reach consumers within one week from harvest, and tomato prices can widely fluctuate, making production planning and harvest scheduling difficult for farmers (39). Smallholder farmers have higher transaction costs, in part because of low economies of scale and low bargaining power (42). Farmer producer organizations may reduce these costs and address some of the disadvantages of smallholder farmers. Cooperative and contract farming may shift the risk of price fluctuations away from farmers to retailers (39). Indian farmers might also benefit from access to accurate price information and clear grading standards using the National Agriculture Market (eNAM), India's electronic trading platform that aims to connect APMCs into a central market. However, eNAM only connects 9% of APMC markets, with slow uptake and usage (42).

Compared to staple crops, the quality of nonstaple crops is more variable (35). While instrumental measurements reduce variation between supply chain actors (43), Indian fruit and vegetable wholesale market services and infrastructure remain basic, with limited use of modern technology and methods to identify and communicate quality differences (35). The eNAM platform indicates commodity quality parameters for fresh fruits and vegetables traded in the wholesale markets, such as the presence of defects, discoloration, the presence of physical injuries, and fruit size. It remains to be seen how quality parameters will be measured and communicated in practice. Further, desirable product attributes for fresh markets and processing markets may differ. Tomato varieties intended for fresh markets are typically evaluated on appearance, taste, and handling, whereas varieties for processing are evaluated on viscosity and soluble solids (44). In the Chittoor district, we observed tomato traders from juice factories purchasing tomatoes, usually from the lowest quality and price lots. Several “dual purpose” tomato varieties have been developed for fresh and processing uses, but they are not yet commercially available (45).

Certain quality attributes, including bruised or cracked skin, evidence of disease or decay, and sun blisters, can render the vegetable inedible (46). Sorting out damaged produce is important to reduce the potential for contamination, reduce the risk of further decay, and ensure the food is edible (47). The South American tomato leaf miner, *Tuta absoluta*, is an invasive pest in India, including in Andhra Pradesh, where the Chittoor district has heavy pest infestations (48). In addition to affecting tomato plant growth, *Tuta absoluta* larvae enter and feed on tomato fruit, causing damage to harvested tomatoes (49). In South India, farmers rely on heavy chemical pesticide applications that may leave residue on the tomato fruit sold to consumers, depending on the last treatment date (48, 50). FLW reduction efforts must balance observable and unobservable quality attributes to ensure the availability of safe food.

This study has several strengths and limitations. Using direct FLW measurement approaches across major supply chain stages with short recall periods, we minimized the time between harvest and market activities and measurement. We surveyed farmers at several harvests on the same plot to account for the multiple tomato harvests in a single season. The findings demonstrate that summary, single-point estimates may obscure FLW patterns for perishable indeterminate crops. Further, loss estimates depend on the data collection method and FLW definition applied, but not at all supply chain stages. Although this study was set in a high-tomato-producing region of India, nearly half of enrolled farm households did not harvest tomatoes. The lower sample size limits generalizability. In the era of climate extremes, water scarcity, which is the reported major production challenge, will likely continue to interrupt production. Capturing field losses for multipicking crops is challenging. Each survey captured field losses on days that farmers had already decided to harvest, therefore missing field losses in between harvests or when harvests were stopped altogether. Future work on FLW measurement methods should consider how and when to capture field losses of market-ready produce for multipicking crops, many of which are fruits and vegetables. We did not collect data on farmer producer organization performance or the level of household farmer producer organization participation, which may have revealed associations with FLW.

More research on perishable produce supply chains is needed, using direct measurement approaches across multiple supply chains stages; exploring the final uses of diverted products and their costs or benefits; and integrating food quality assessments with FLW estimates. Such evidence can inform on opportunities for FLW reduction policies and initiatives aiming to create sustainable food systems that promote positive health and environmental outcomes. Strategies to reduce FLW should also focus on the underlying reasons for loss, including postharvest handling, seasonality, and market structures.

## Supplementary Material

nqac039_Supplemental_File

## Data Availability

Data described in the manuscript, code book, and analytic code will be made available upon request.

## Notes

This study was funded by the Bill and Melinda Gate's Foundation, Seattle, WA (Investment INV-006624; Technical Assistance and Research for Indian Nutrition and Agriculture).

The funding agency was not involved in the study design; collection, analysis, and interpretation of the data; writing and preparation of the manuscript; and decision to submit the manuscript for publication.

Supplemental Tables 1–5, Supplemental Methods, and Supplemental Figures 1–3 are available from the “Supplementary data” link in the online posting of the article and from the same link in the online table of contents at https://academic.oup.com/ajcn/.

Abbreviations used: APMC, Agricultural Produce Market Committee; eNAM, National Agriculture Market; FLW, food loss and waste; SDG, Sustainable Development Goal.
