# Supplementary material for: Food loss of perishable produce from farm to retail: evidence from tomato supply chains in South India
Source: Am J Clin Nutr. 2022 Feb 14;115(6):1535–48. doi: 10.1093/ajcn/nqac039 (PMC9170466; doi:10.1093/ajcn/nqac039)
Supplement: nqac039_Supplemental_File [file nqac039_supplemental_file.docx]

Online Supplementary Material

**Title:**

Food loss of perishable produce from farm to retail: evidence from tomato supply chains in South India

**Authors:**

Jocelyn M. Boiteau; Prabhu Pingali

**Author affiliations:**

Tata-Cornell Institute for Agriculture and Nutrition, Cornell University, Ithaca, New York, USA (JMB, PP)

Division of Nutritional Sciences, Cornell University, Ithaca, New York, USA (JMB, PP)

Charles H. Dyson School of Applied Economics and Management, Cornell University, Ithaca, New York, USA (PP)

**Corresponding author**:

Jocelyn M. Boiteau

Tata-Cornell Institute for Agriculture and Nutrition, Cornell University

375 Warren Hall, Ithaca, NY 14853, USA

[jmb575@cornell.edu](mailto:jmb575@cornell.edu)

1. Supplementary methods: Rationale for independent variable selection for determinants of loss

Regarding household characteristics, longer production experience, higher education levels, and agriculture as a main income source have been found to be significantly associated with decreased food loss at the farmer stages (1). To account for education level, we used the highest education level attained from a surveyed household member, rather than the education level of the household head, because a household head’s characteristics may be insufficient to characterize the household and all household decision-makers (2). We assume that surveyed household members responsible for the harvesting and marketing aspects of tomato production are decision-makers.

At the plot-level, production activities and inputs that occurred once per plot are applied to all harvests of that plot. Production activities and inputs that occurred at different time-points on the plot were accounted for at the harvest-level. Plot-level activities and inputs included sapling density, growing area, and the tomato breed planted. Harvest-level activities and inputs included types of irrigation, staking and plastic sheeting, and fertilizer and fungicide applications. Production activities, from choice of breed and planting to irrigation practices and fertilizer applications, impact the fruit quality and can reduce product degradation before and after harvest (3,4). With regard to harvest number, previous studies have demonstrated that, for each plot, the vegetables from the first or second harvest are the best quality because the plants are young and healthy compared to later on in the season where the plants lose vigor and harvest traffic causes some damage (5). Harvest, post-harvest and marketing factors may influence food loss, including harvest timing and tomato quality; harvest and post-harvest labor and handling; and transportation and marketing conditions (3,4,6–9).

In addition to hired and family labor, we accounted for labor gender to understand the involvement of females and males as the main actor in a particular supply chain step (10). Finally, we explored participant-reported production and marketing limitations, as well as loss reduction strategies and information sources. Previous research demonstrates higher levels of food loss when producers reported production limitations such as animals, pests and disease (1). Inadequate knowledge or information from production to marketing stages may also increase food loss (6).

1. Supplementary methods: Equations used in two-part models for each food loss outcome
2. Mixed effects logit model

$log[\frac{P_{hpfv}}{1-P_{hpfv}}] = \beta_{0}+\beta_{1}X_{\mathrm{hpfv}} + \beta_{2}X_{\mathrm{pfv}}+ \beta_{3}X_{\mathrm{fv}} + \beta_{4}X_{v} + \varepsilon_{v}+ \varepsilon_{\mathrm{fv}}+ \varepsilon_{\mathrm{pfv}}+ \varepsilon_{\mathrm{hpfv}}$

1. Mixed effects linear model

ln[$Y_{hpfv}|Y_{hpfv}>0] = \beta_{0}+\beta_{1}X_{\mathrm{hpfv}} + \beta_{2}X_{\mathrm{pfv}}+ \beta_{3}X_{\mathrm{fv}} + \beta_{4}X_{v} + \varepsilon_{v}+ \varepsilon_{\mathrm{fv}}+ \varepsilon_{\mathrm{pfv}}+ \varepsilon_{\mathrm{hpfv}}$

*P_hpfv_* is the probability of a positive food loss outcome at harvest *h*, on plot *p*, of farm household *f*, in village *v*

Y_hpfv_ | Y_hpfv_ >0 is the food loss estimate conditional on a positive outcome at harvest *h*, on plot *p*, of farm household *f*, in village *v*

β_0_ is the constant

β_1_X_hpfv_ are the estimated coefficients of observed characteristics of harvest *h*, on plot *p*, of farm household *f*, in village *v*

β_2_X_pfv_ are the estimated coefficients of observed characteristics of plot *p*, of farm household *f*, in village *v*

β_3_X_fv_ are the estimated coefficients of observed farm household characteristics *f*, in village *v*

β_4_X_v_ are the estimated coefficients of observed characteristics of village *v*

ε are the unobserved disturbance terms across village variation, across farm household variation within villages, across plot variation within farm households within villages, and across harvest variation within farm households within villages

Supplemental Table 1 Definition of variables and measurement used in modeling determinants of food loss

| **Groupings** | **Variable** | **Type** | **Definition and measurement** |
| --- | --- | --- | --- |
| **Outcome variables** |  |  |  |
|  | Pre-harvest damage | Dummy | = 1 if pre-harvest damage is >0% |
|  | Extent of pre-harvest damage | Continuous | Share of harvest with pre-harvest damage among harvests with pre-harvest damage >0% |
|  | Post-harvest, farm-level food loss | Dummy | = 1 if post-harvest, farm level food loss is >0% |
|  | Extent of post-harvest, farm-level food loss | Continuous | Share of harvest with post-harvest, farm level food loss among harvests with post-harvest, farm level food loss >0% |
|  | Pre-auction, market-level food loss | Dummy | = 1 if pre-auction, market level food loss is >0% |
|  | Extent of pre-auction, market-level food loss | Continuous | Share of harvest with pre-auction, market-level food loss among harvests with pre-auction, market level food loss >0% |
| **Covariate variables** |  |  |  |
|  | Local NGO | Dummy | = 1 if *panchayat* falls under local non-governmental (NGO) program area |
|  | Caste | Dummy | = 1 if household is SC, ST or BC |
| **Independent variables** |  |  |  |
| Socio-economic | Education | Dummy | = 1 if household member respondent highest education is at least secondary (grade 8) |
|  | Income source | Dummy | = 1 if household primary income from agriculture |
|  | Experience | Continuous | Number of years' experience in tomato production |
|  | FPO | Dummy | = 1 if household is a member of a Farmer Producer Organization (FPO) |
|  | Land owned, acres | Continuous | Number of acres of land household owns |
|  | Land leased, acres | Continuous | Number of acres of land household leases |
| Plot-level production | Sapling density | Continuous | Number of saplings (100) per acre |
|  | Grow area | Continuous | Number of acres planted |
|  | PHS 448 breed | Dummy | = 1 if planted PHS 448 breed |
|  | Saaho breed | Dummy | = 1 if planted Saaho breed |
|  | US 440 breed | Dummy | = 1 if planted US 440 breed |
|  | Other breed used | Dummy | = 1 if planted breed other than PHS 448, Saaho, or US 440 |
| Harvest-level production | Drip irrigation | Dummy | = 1 if used drip irrigation |
|  | Canal irrigation | Dummy | = 1 if used canal irrigation |
|  | Staking | Dummy | = 1 if used staking and twine |
|  | Plastic sheeting | Dummy | = 1 if used plastic sheeting |
|  | Chemical fertilizer applied | Dummy | = 1 if used chemical fertilizer |
|  | Farmyard manure applied | Dummy | = 1 if used farmyard manure |
|  | Fungicide applied | Dummy | = 1 if used fungicide |
| Decision to harvest | Harvest number | Continuous | Number of harvest for the production area in current harvesting season |
|  | Peak harvest season | Dummy | =1 if harvest during peak season (April-July) |
|  | Max. market price expected, at farm-level | Continuous | Maximum price per 30 kg farmer expects to receive at auction (100 Rs.), reported at farm-level on day of harvest |
| Decision to harvest, quality | Harvest based on ripeness level | Dummy | =1 if major reason for harvesting was the ripeness level |
|  | Maximum ripeness level | Categorical | = 0 if maximum ripeness level is pink/light red |
|  |  |  | = 1 if maximum ripeness level is red |
|  |  |  | = 2 if maximum ripeness level is super red |
|  | Harvest quality intensity | Continuous | Overall quality intensity of harvest from low to high quality (scale 1-9) |
| Harvest labor | Harvest labor: hired, female | Dummy | = 1 if hired, female participated in harvesting |
|  | Harvest labor: hired, male | Dummy | = 1 if hired, male participated in harvesting |
|  | Harvest labor: family, female | Dummy | = 1 if family, female participated in harvesting |
|  | Harvest labor: family, male | Dummy | = 1 if family, male participated in harvesting |
|  | Harvest labor: family, female child | Dummy | = 1 if family, female child participated in harvesting |
|  | Harvest labor: family, male child | Dummy | = 1 if family, male child participated in harvesting |
| Harvest handling | Harvest container: basket | Dummy | = 1 if harvesting container is a basket |
|  | Harvest container: plastic crate | Dummy | = 1 if harvesting container is a plastic crate |
|  | Harvest container: plastic bucket | Dummy | = 1 if harvesting container is a plastic bucket |
|  | Harvest container: aluminum bucket | Dummy | = 1 if harvesting container is an aluminum bucket |
| Post-harvest handling | Harvest total | Continuous | Total amount of harvested tomatoes (30 kg) |
|  | Field shade | Dummy | = 1 if harvested tomatoes are kept in a shaded area at the field |
|  | Field container | Categorical | = 0 if no container used to hold harvested tomatoes at the field |
|  |  |  | = 1 if crate ≤ 20 kg container used to hold harvested tomatoes at the field |
|  |  |  | = 2 if crate ≥ 25 kg container used to hold harvested tomatoes at the field |
|  | Release field head | Dummy | = 1 if release field heat from tomatoes after harvest |
|  | Farm-level grading | Dummy | = 1 if grading done at the farm-level |
|  | Grading done before transport to market | Dummy | = 1 if grading done before transport to market |
| Preparing for market | Aggregate | Dummy | = 1 if farmer aggregated with other farmers to transport tomatoes to market |
|  | Packing labor: hired, female | Dummy | = 1 if hired, female participated in packing |
|  | Packing labor: hired, male | Dummy | = 1 if hired, male participated in packing |
|  | Packing labor: family, female | Dummy | = 1 if family, female participated in packing |
|  | Packing labor: family, male | Dummy | = 1 if family, male participated in packing |
|  | Madanapalle market | Dummy | = 1 if farmer plans to sell tomatoes at Madanapalle tomato wholesale market |
| Farm level quality limitations | Production limitation: climate | Dummy | = 1 if climate is a problem to producing quality tomatoes |
|  | Production limitation: insects/disease | Dummy | = 1 if insect/disease is a problem to producing/marketing quality tomatoes |
|  | Production limitation: Price fluctuations | Dummy | = 1 if price fluctuations is a problem to producing/marketing quality tomatoes |
|  | Production limitation: Poor transport | Dummy | = 1 if poor transport is a problem to producing/marketing quality tomatoes |
|  | Production limitation: Cost of inputs | Dummy | = 1 if cost of inputs is a problem to producing/marketing quality tomatoes |
| Farm level loss strategies | Loss reduction strategy: Apply pesticides | Dummy | = 1 if applying pesticides is a loss reduction strategy |
|  | Loss reduction strategy: Careful harvest handling | Dummy | = 1 if careful handling during harvest is a loss reduction strategy |
| Market transport | Market transport, hired | Dummy | = 1 if use hired transporter to bring tomatoes to market |
|  | Transport day | Dummy | = 1 if tomatoes transported to market day after harvest |
|  | Transport during daylight | Dummy | = 1 if tomatoes transported during daylight hours |
|  | Unloading labor: hired, female | Dummy | = 1 if hired, female participated in unloading tomatoes at market |
|  | Max. number of crates stacked | Continuous | Maximum number of crates (filled with tomatoes) stacked together |
| Market grading | Market grading covered | Dummy | = 1 if market grading is done in a covered area |
|  | Market grading labor: hired, female | Dummy | = 1 if hired, female participated in market grading |
|  | Market grading labor: hired, male | Dummy | = 1 if hired, male participated in market grading |
|  | Market grading labor: family, female | Dummy | = 1 if family, female participated in market grading |
|  | Market grading labor: family, male | Dummy | = 1 if family, male participated in market grading |
|  | Max. market price expected, at market-level | Continuous | Maximum price per 30 kg farmer expects to receive at auction (100 rs.), reported at market-level |
| Marketing limitations | Marketing limitation: Climate | Dummy | = 1 if climate is a problem for marketing quality tomatoes |
|  | Marketing limitation: Price fluctuations | Dummy | = 1 if price fluctuations are a problem for marketing quality tomatoes |
|  | Marketing limitation: Poor transport | Dummy | = 1 if poor transport is a problem for marketing quality tomatoes |
|  | Marketing limitation: Transport costs | Dummy | = 1 if transport costs are a problem for marketing quality tomatoes |
| Production information sources | Production: other farmers | Dummy | = 1 if other farmers are a source for production information |
|  | Production: agricultural officers | Dummy | = 1 if agricultural officers are a source for production information |
|  | Production: input suppliers | Dummy | = 1 if input suppliers are a source for production information |
|  | Production: family | Dummy | = 1 if family are a source for production information |
| Post-harvest information sources | Post-harvest: other farmers | Dummy | = 1 if other farmers are a source of post-harvest information |
|  | Post-harvest: tomato traders/mandi owners | Dummy | = 1 if tomato traders/mandi owners are a source of post-harvest information |
|  | Post-harvest: family | Dummy | = 1 if family are a source of post-harvest information |
| Grading information sources | Grading: tomato traders/mandi owners | Dummy | = 1 if tomato traders/mandi owners are a source of grading information |
|  | Grading: farmer, themselves | Dummy | = 1 if the farmer's own experience is a source of grading information |
|  | Grading: family | Dummy | = 1 if family are a source of grading information |
|  | Grading: laborers/market laborers | Dummy | = 1 if laborers/market laborers are a source of grading information |
| Price information sources | Price: other farmers | Dummy | = 1 if other farmers are a source of price information |
|  | Price: tomato traders/mandi owners | Dummy | = 1 if tomato traders/mandi owners are a source of price information |
|  | Price: phone/online messaging groups | Dummy | = 1 if phone/online messaging groups are a source of price information |
|  | Price: transporters | Dummy | = 1 if transporters are a source of price information |
| Commission agent choice | Agent: provides credit | Dummy | = 1 if farmer chooses agent because agent provides credit |
|  | Agent: on-time payment | Dummy | = 1 if farmer chooses agent because agent gives payments on-time |
|  | Agent: trustful | Dummy | = 1 if farmer chooses agent because agent is trustful, overall |
|  | Agent: fixes rate | Dummy | = 1 if farmer chooses agent because agent fixes the auction rate |
|  | Agent: provides crates | Dummy | = 1 if farmer chooses agent because agent provides plastic crates |

**Supplemental Table 2** Tomato trader, vegetable trader, and vegetable retailer characteristics^1^

|  |  | Tomato trader |  |  | Vegetable trader |  |  | Vegetable retailer |
| --- | --- | --- | --- | --- | --- | --- | --- | --- |
|  | n | Median (IQR) |  | n | Median (IQR) |  | n | Median (IQR) |
| Surveys per participant | 83 | 1 (1, 2)^2^ |  | 52 | 8 (6, 9) |  | 50 | 7 (5, 8) |
| Male participant, *n (%)* | 83 | 83 (100) |  | 52 | 52 (100) |  | 50 | 40 (80) |
| Education level ≥ grade 8, *n (%)* | 83 | 64 (77) |  | 51 | 47 (90) |  | 50 | 23 (46) |
| Experience in tomato business, *years* | 83 | 15 (6, 20) |  | 51 | 19 (10, 27) |  | 49 | 15 (8, 25) |
| Retail type, *n(%)* |  | - |  |  | - |  | 50 |  |
| Daily market |  |  |  |  |  |  |  | 35 (70) |
| Local brick and mortar |  |  |  |  |  |  |  | 4 (14) |
| Weekly market |  |  |  |  |  |  |  | 4 (8) |
| Pushcart/roadside shop |  |  |  |  |  |  |  | 4 (8) |

^1^ Sample size (n) changes by row due to data availability, reported values are median (IQR), unless otherwise indicated

^2^ Surveys per tomato trader were skewed. Min. = 1, Max. = 19. Mean (SD) = 2.4 (3.3) surveys

**Supplemental Table 3** Food loss and waste estimates by declared and destination loss methods at wholesale and retail stages^1^

|  | **Declared FLW^2^** | | |  | **Destination FLW^3^** | | |
| --- | --- | --- | --- | --- | --- | --- | --- |
|  | n | Mean ± SD^4^ | Median (IQR) |  | n | Mean ± SD | Median (IQR) |
| **Tomato wholesale FLW (post-auction)^5^** |  |  |  |  |  |  |  |
| Frequency of lots with FLW, *n (%)* | - | - | - |  | 60 | 4 (7) |  |
| Among all lots, share of lots lost, *%* |  |  |  |  |  | 0.0 (0.1) | 0.0 (0.0, 0.0) |
| Among lots with loss, share of lots lost, *%* |  |  |  |  |  | 0.4 (0.5) | 0.3 (0.2, 0.7) |
| **Vegetable wholesale FLW** |  |  |  |  |  |  |  |
| Frequency of lots with FLW, *n (%)* | 182 | 0 (0) |  |  | 78 | 46 (59) |  |
| Among all lots, share of lots lost, *%* |  | 0.0 (0.0) | 0.0 (0.0, 0.0) |  |  | 0.7 (2.3) | 0.2 (0.0, 0.7) |
| Among lots with loss, share of lots lost, *%* |  | - | - |  |  | 1.3 (3.0) | 0.5 (0.2, 1.0) |
| **Vegetable retail FLW** |  |  |  |  |  |  |  |
| Frequency of lots with FLW, *n (%)* | 110 | 97 (88) |  |  | 331 | 87 (26) |  |
| Among all lots, share of lots lost, *%* |  | 4.2 (4.9) | 2.9 (1.0, 5.0) |  |  | 1.9 (7.4) | 0.0 (0.0, 0.4) |
| Among lots with loss, share of lots lost, *%* |  | 4.8 (4.9) | 3.6 (1.6, 6.0) |  |  | 7.1 (13.2) | 2.6 (1.5, 6.2) |

^1^ Sample size (n) changes by row due to data availability; based on field observations, common reasons for missing data were the participant did not know or the participant was not available to complete all survey questions.

^2^ Tomato wholesale loss: FLW estimated using participant self-report. Vegetable wholesale loss: estimated using participant reported inventory received and sold. Vegetable retail loss: participant-reported FLW from previous day, at the end of selling.

^3^ Tomato wholesale and vegetable wholesale loss: FLW estimated using participant-reported crate counts and considering loss destinations as any, non-food use. Vegetable retailer loss: FLW estimated using crate counts and considering loss destinations as any, non-food use at start of sales on survey day.

^4^ Values are mean ± SD unless otherwise indicated

^5^ Due to skewed number of surveys per tomato trader, loss estimates are collapsed to the mean per trader.

Note: abbreviations: FLW, food loss and waste

**Supplemental Table 4** Summary statistics for independent variables included in final models of determinants of loss^1^

| Variable | n | Mean ± SD | Median (IQR) |
| --- | --- | --- | --- |
| Production input: drip irrigation, *n (%)*^2 3^ | 276 | 229 (83) | - |
| Production input: staking, *n (%)*^2 3^ | 276 | 249 (90) | - |
| Production input: chemical fertilizer or NPK applied, *n (%)*^2 3^ | 276 | 252 (91) | - |
| Harvest number^2^ | 276 | 4.59 ± 2.92 | 4 (3, 6) |
| Harvest season, peak season (April-July), *n (%)*^2^ | 276 | 160 (58) | - |
| Highest price expected, *100 Rs. per 30 kg*^2^ | 247 | 5.12 ± 2.47 | 5.5 (3.0,7.0) |
| Quality intensity, *low to high quality (1-9)*^2^ | 275 | 5.83 (2.19) | 6.08 (4.65,7.74) |
| Loss reduction strategy: applied pesticides, *n (%)*^2 3^ | 275 | 70 (25) | - |
| Total harvested tomatoes, *30 kg*^2^ | 264 | 25.71 ± 26.25 | 17 (9,33) |
| Area tomatoes kept during harvest: shaded, *n (%)*^2 3^ | 276 | 191 (69) |  |
| Container used to hold harvested tomatoes at the field, *n (%)*^2^ | 276 |  |  |
| No container |  | 20 (7.3) | - |
| Plastic crate, ≤ 20 kg capacity |  | 34 (12) | - |
| Plastic crate, ≥ 25 kg capacity |  | 222 (80) | - |
| Grading and sorting done on-farm, *n (%)*^2 3^ | 274 | 133 (48) | - |
| Harvesting container is a basket, *n (%)*^2 3^ | 275 | 67 (24) | - |
| Farm-level packing, family, male, *n (%)*^2 3^ | 275 | 224 (81) | - |
| Farm-level packing, hired, female, *n (%)*^2 3^ | 275 | 87 (32) | - |
| Market-level grading, family, male, *n (%)*^3 4^ | 186 | 113 (61) | - |
| Market-level grading, hired, female, *n (%)*^3 4^ | 186 | 100 (54) | - |

^1^ Sample size (n) changes by row due to data availability; based on field observations, common reasons for missing data were the participant did not know or, particularly at the market, the participant was not available to complete all survey questions. Reported values are mean ± SD, unless otherwise indicated.

^2^ Data collected from farm level surveys

^3^ Dummy, yes/no variable; summary statistics refer to "yes"

^4^ Data collected from market level surveys

Note: abbreviations: NPK, nitrogen, phosphorus, and potassium; Rs., Indian Rupees

**Supplemental Table 5** Pile sort grouping domains, categories and aspects

| Grouping domain | Grouping category | Specific grouping aspects reported by participants |
| --- | --- | --- |
| Quality grade | Market grade | 1^st^ quality/A grade; 2^nd^ quality; B grade; 3^rd^ quality/C grade; 4^th^ quality/D grade; Damaged |
|  | Quality group | High; good; medium; low; last |
| Quality attribute | Size | Big; medium; small |
|  | Color/ripeness | Over-ripe; Ripened/red-ripe; semi-ripened; unripe/green; shine; discolored |
|  | Firmness | Firm; soft |
|  | Storage performance | Can be stored; cannot be stored/consume same-day |
|  | Transport performance | Good for transport; not good for transport |
|  | Pest/disease/physical damage | Black spots; oozi; worms; broken; leaking; spoiled/rotten; holes; blisters |
| Use/destination | Use | Fresh food; processing; animal feed; discard |
|  | Destination | Market; household; leave on vine/field; trash; no destination |
| Marketability | Always sell | Sell at high or low rates |
|  | Sometimes sell | Sell at high rates, cannot sell at low rates |
|  | Never sell | Cannot sell at any rate |

**
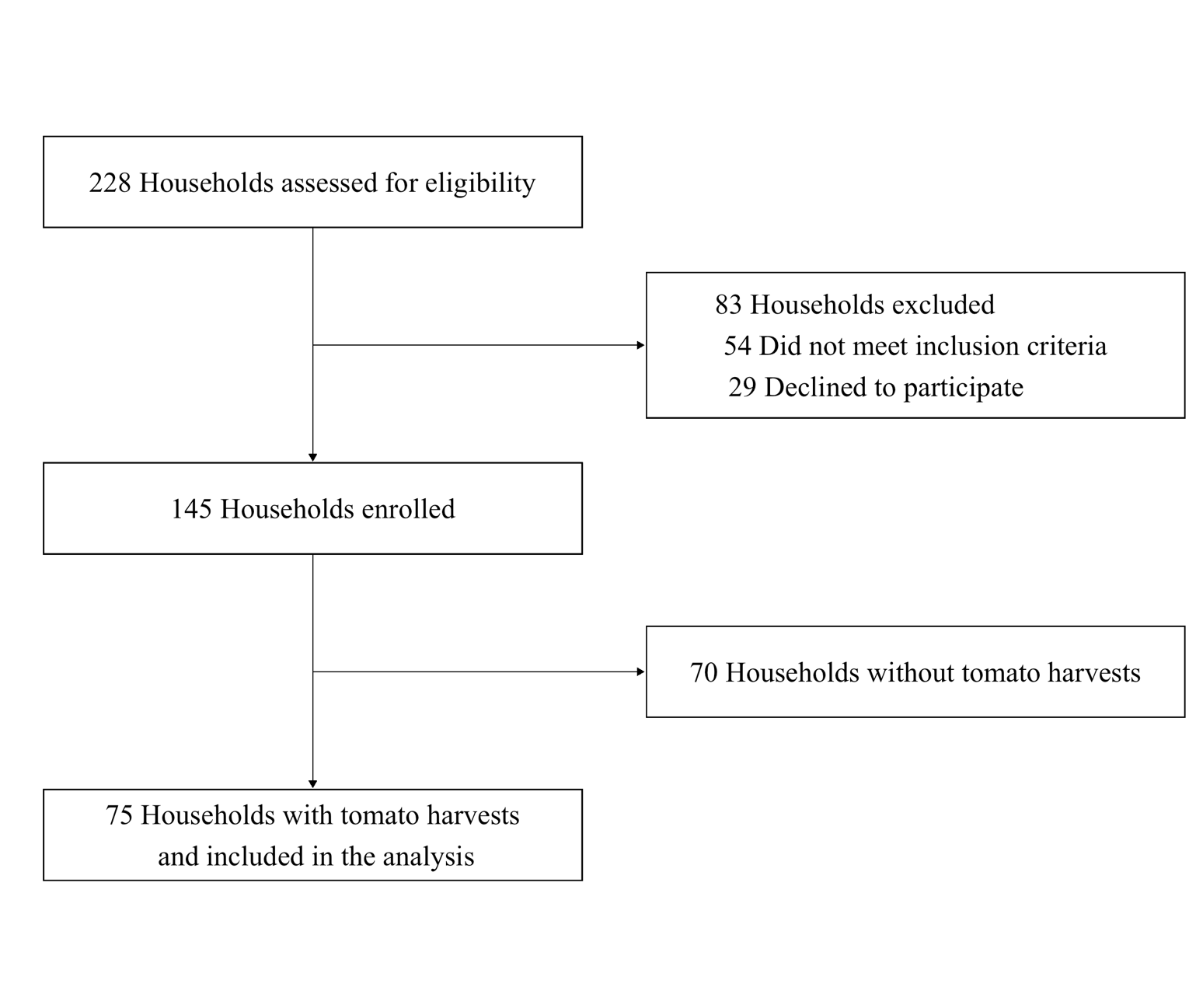
Supplemental Figure 1.** Farm household participant flow chart

**
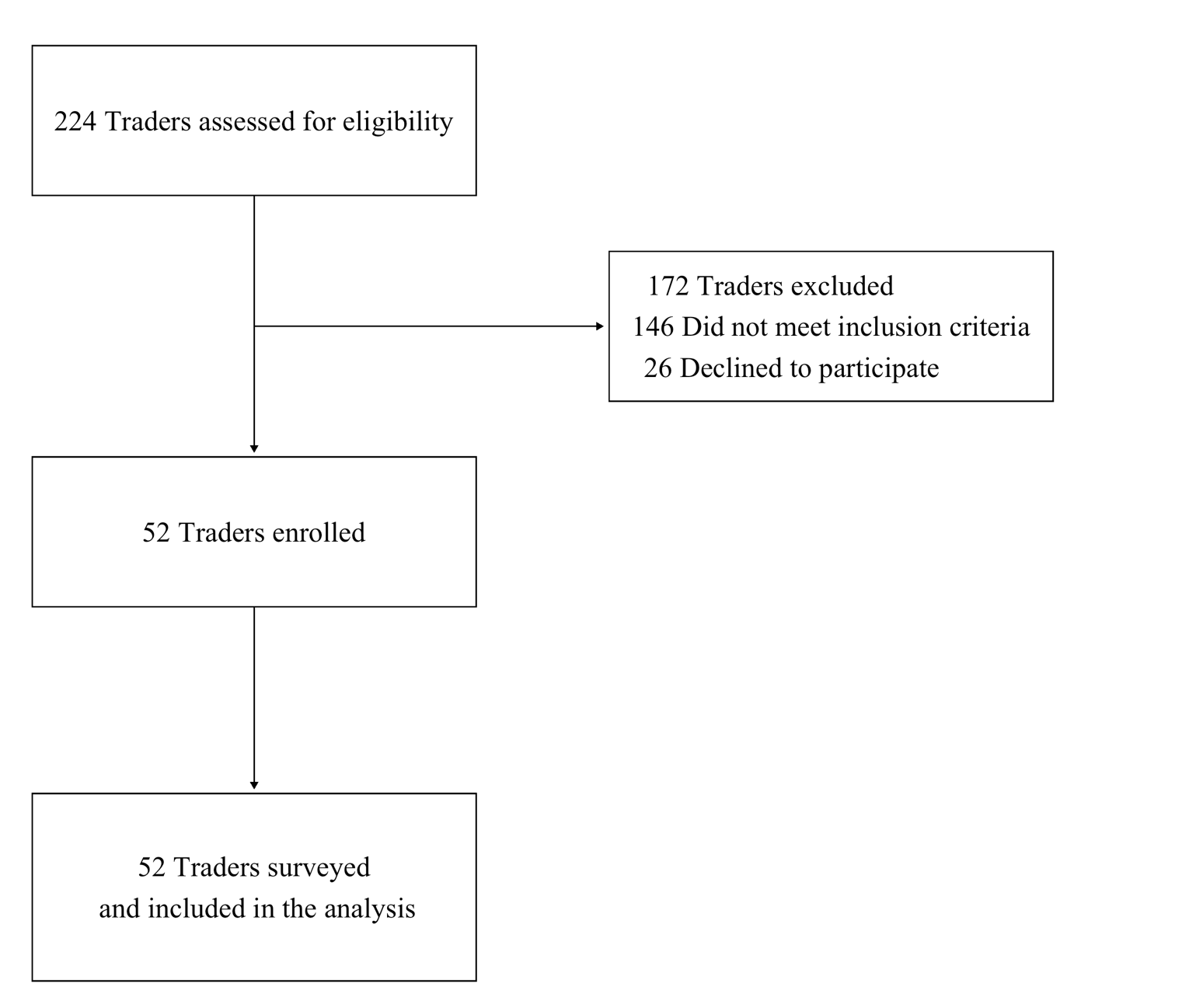
Supplemental Figure 2.** Vegetable trader participant flow chart

**
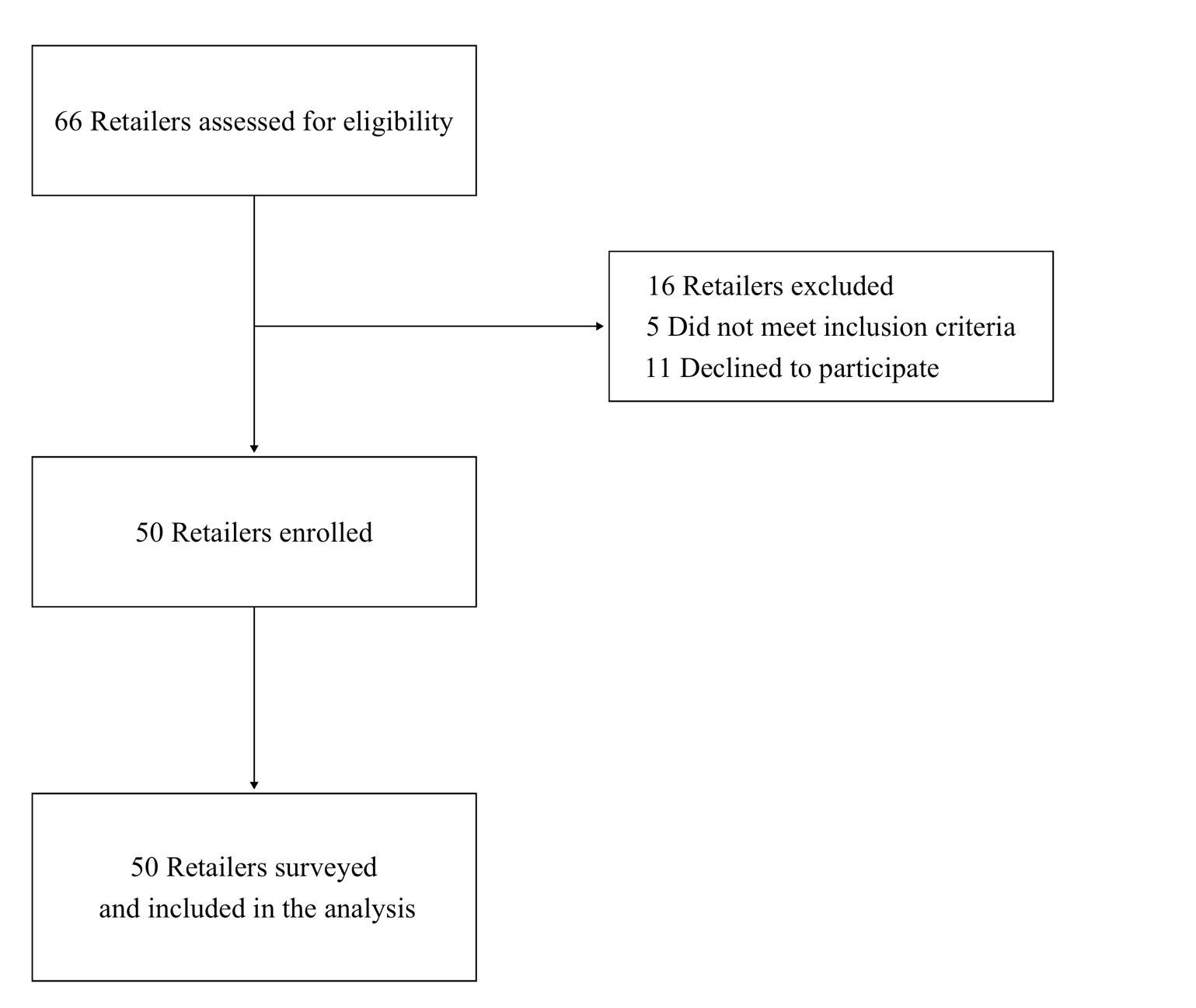
Supplemental Figure 3.** Vegetable retailer participant flow chart

**References**

1. Delgado L, Schuster M, Torero M. Reality of Food Losses: A New Measurement Methodology. 2017.

2. Kleinjans KJ. The man of the house-How the use of household head characteristics may lead to omitted variable bias. Econ Lett Elsevier B.V.; 2013;119:133–5.

3. Kitinoja L, Kader A. Small-Scale Postharvest Practices A Manual for Horticultural Crops - Postharvest Technology Center. 2002.

4. Kader AA, Rolle RS. The role of post-harvest management in assuring the quality and safety of horticultural produce. FAO Agricultural Services Bulletin. Rome; 2004.

5. Dunning R, Johnson L, Boys K. Putting Dollars to Waste: Estimating the Value of On-Farm Food Loss. Choices 2019;34:1–9.

6. Rolle RS, editor. Reports of the APO seminar on Reduction of Postharvest Losses of Fruit and Vegetables held in India, 5–11 October 2004 and Marketing and Food Safety: Challenges in Postharvest Management of Agricultural/Horticultural Products in Islamic Republic of Iran, . Rome; 2006.

7. Magalhães VSM, Ferreira LMDF, Silva C. Using a methodological approach to model causes of food loss and waste in fruit and vegetable supply chains. J Clean Prod 2021;283.

8. Mohammed M, Craig K. Food loss analysis : causes and solutions Case study on the tomato value chain in the Republic of Guyana. 2018.

9. Kader AA. Handling of horticultural perishables in developing vs. developed countries. Acta Hortic 2010;877:121–6.

10. FAO. Gender and food loss in sustainable food value chains - A guiding note. 2018.
